# Pipeline Flange Bolt Loosening Detection Technology Based on Stress Waves and Deep Learning

**DOI:** 10.3390/s26103120

**Published:** 2026-05-15

**Authors:** Cong Yu, Peng Cheng, Chenxi Shao, Yehang Guo, Lu Cheng, Chao Sun

**Affiliations:** China Productivity Center for Machinery Co., Ltd., China Academy of Machinery Science and Technology, Beijing 100044, China; yc010618@163.com (C.Y.);

**Keywords:** vibrations, pipe flange, bolt, looseness detection, stress wave, SVM, RFE

## Abstract

Flanged connections are a critical joining method in modern industrial production, making the detection of bolt loosening in flanges a vital step to ensure industrial safety. Current research on bolt loosening detection in flanges mainly focuses on flat-face flanges without gaskets, while studies on bolted pipe flanges containing gaskets are relatively limited. To achieve bolt loosening detection in such gasketed pipe flanges, this paper analyzes the influence of bolt loosening on wave propagation in the gasket based on the stress wave principle and finite element simulation, and employs the hammer impact method to realize the detection of bolt loosening degree in pipeline flanges. The optimal knock force and hammer head material for the bolt loosening detection experiments were determined experimentally. Through comparative experiments, the Support Vector Machine—Recursive Feature Elimination (SVM-RFE) model was identified as being more accurate and efficient in assessing the degree of bolt loosening. Furthermore, the model was optimized by incorporating feature enhancement and cost-sensitive learning, thereby providing a reliable methodological solution for the rapid identification of bolt loosening severity in pipeline flanges.

## 1. Introduction

In industrial production and modern engineering systems, bolted flange connections, serving as a fundamental and critical component joining method, are extensively utilized in various sealing and load-bearing structures. Ranging from large-scale petrochemical storage tanks [1], oil pipelines [2], and pressure vessels in nuclear power plants [3], to aero-engines [4,5], wind energy applications [6,7,8], and high-speed train bogie connections [9,10], bolted flange connections have become the structural core for joining components due to their advantages such as low cost, high reliability, detachability, excellent sealing performance, and strong load-bearing capacity [11,12]. However, in actual operating environments, due to the influence of multiple complex conditions including vibration, thermal cycling expansion, material creep, and uneven initial installation preload, bolts inevitably face the risk of loosening, preload decay, or even detachment, seriously threatening system integrity and personnel safety [13,14,15]. Consequently, detecting bolt loosening in flanges constitutes an essential means of ensuring system reliability.

The traditional detection method relies on manual inspection, utilizing manual tapping, wrenches, or visual observation to assess loosening. While manual inspection allows for the individual checking of numerous bolts on a flange and timely preload correction, this method suffers from disadvantages such as substantial workload and time consumption. Furthermore, due to the subjective nature of manual inspection, issues such as errors or missed detections may arise. With the advancement of detection technology, techniques including visual inspection [16,17], piezoelectric impedance detection [18], and ultrasonic testing [19,20] have been applied in the field of bolt loosening detection. Currently, scholars worldwide have conducted extensive research in this domain. Xu et al. [21] proposed an improved time-reversal method for monitoring bolt loosening, extracting the phase shift and signal amplitude of the focused wave packet in the reconstructed signal as a tightness index to identify bolt loosening. Huo et al. [22], based on the electromechanical impedance method, proposed a technique utilizing a “smart washer” for detecting bolt preload loosening, evaluating loosening criteria through changes in the root mean square deviation of the impedance signal. Addressing the issue of bolt loosening in offshore wind turbines, Wang et al. [23] proposed a detection method employing vibration sensors and CCD sensors, determining bolt status by identifying changes in prefabricated marks on the bolts. However, this method fails to achieve multi-bolt detection.

Recently, to address the issues of data distribution shift and scarcity of labeled samples in fault diagnosis, researchers have proposed various domain generalization and few-shot learning strategies. For instance, Chen et al. [24] proposed a shrinkage Mamba relation network (SMRN), which generates out-of-distribution data (OODD) through soft Brownian offset. Combined with a residual shrinkage module and a Mamba relation module, this method achieves accurate fault detection and localization in rotating machinery under zero-faulty data conditions. By constructing feature pairs between health data and OODD, this approach effectively reveals inter-class unique attributes and intra-class inherent attributes, offering new insights for feature extraction in small-sample scenarios. Furthermore, Chen et al. [25] proposed a learning category-invariant disentangled features (LCIF) framework. Different from traditional domain alignment paradigms, this framework explicitly decomposes machine states into domain attributes reflecting operating conditions and category attributes reflecting fault types. Through disentanglement learning, it directly extracts category-invariant features that are essentially related to faults, achieving significant improvements in generalization performance for cross-condition fault diagnosis tasks. The above works provide valuable references for handling data distribution differences, feature disentanglement, and limited sample issues.

Current research on bolt loosening in flanges predominantly focuses on flat-face contact flanges without gaskets, with the detection principle based on the contact stiffness between the flange faces. However, for flanges sealed with gaskets, achieving an analysis of the nonlinear elastic properties of the gasket proves challenging; studies specifically addressing bolt loosening in sealed pipeline flanges are relatively scarce [26].

To fill the technical gap in bolt loosening detection for flanges with gaskets and to investigate the feasibility of applying the stress wave-based bolt loosening detection method to sealed pipeline flanges, this paper focuses on a pipeline flange using a spiral wound gasket. The study first analyzes the influence of bolt loosening on stress wave propagation within the system through theoretical methods and finite element simulation. Subsequently, bolt loosening detection experiments are conducted to determine the optimal detection parameters. A multi-domain feature system is constructed, and the experimental results are compared and analyzed to identify the most effective model for detecting bolt loosening. The selected detection model is then optimized through feature enhancement and cost-sensitive learning. The optimized model is used to detect the degree of bolt loosening under different excitation points and varying positions of loosened bolts. The results show that the method still maintains good detection performance when the excitation point and the position of the loosened bolt change.

## 2. Principle of Bolt Loosening Detection Based on Stress Waves

### 2.1. Propagation of Stress Waves

Stress waves are wave forms of stress and strain propagating in an elastic medium. When a material point is disturbed from its equilibrium position by an external force, causing strain, it is driven by internal elastic forces to vibrate. This vibration, in turn, acts as a new disturbance source, transmitting the strain state to surrounding particles through the inherent elastic connections between material points, causing vibration to propagate within the structure. According to different propagation directions, stress waves can be classified into two main types: transverse waves and longitudinal waves. For longitudinal waves, the particle vibration direction is parallel to the wave propagation direction; for transverse waves, the particle vibration direction is perpendicular to the wave propagation direction. The dynamic equation of stress wave propagation in a medium can be represented by the Lamé equation:

(1) $$\left(\lambda +\mu \right)\nabla \left(\nabla \cdot \overset{⃑}{u}\right)+\mu \nabla^{2}\overset{⃑}{u}+\rho \overset{⃑}{F}=\rho \partial^{2}\overset{⃑}{u}/\partial t^{2}$$

where λ and μ are Lamé constants, with μ representing the shear modulus of the material. $\overset{⃑}{u}$ represents the displacement vector field. $\nabla \cdot \overset{⃑}{u}$ represents the divergence of displacement, indicating relative volume change. $\nabla \left(\nabla \cdot \overset{⃑}{u}\right)$ represents the gradient of the displacement divergence. $\nabla^{2}\overset{⃑}{u}$ represents the Laplacian of displacement, describing the “diffusion” or “bending” of the displacement field. $\overset{⃑}{F}$ is the body force per unit volume. ρ is the density of the material.

According to the Helmholtz decomposition, any vector field can be decomposed into an irrotational field (gradient of a scalar potential) and a solenoidal field (curl of a vector potential). Assume the displacement vector:

(2) $$\overset{⃑}{u}=\nabla \phi +\nabla \overset{⃑}{\psi }$$

where ϕ is the scalar potential, related to longitudinal waves. $\overset{⃑}{\psi }$ is the vector potential, related to transverse waves.

Substituting Equation (2) into Equation (1) and neglecting the influence of body forces ($\overset{⃑}{F}=0$), after a series of vector operations, two wave equations can be obtained:

Longitudinal wave equation:

(3) $$\nabla^{2}\phi =\partial^{2}\phi /c_{p}^{2}\partial t^{2}$$

Transverse wave equation:

(4) $$\nabla^{2}\overset{⃑}{\psi }=\partial^{2}\overset{⃑}{\psi }/c_{s}^{2}\partial t^{2}$$

where $c_{p}=\sqrt{\left(\lambda +2\mu \right)/\rho }$ represents the longitudinal wave velocity, and $c_{s}=\sqrt{\mu /\rho }$ represents the transverse wave velocity.

Analysis of Equations (3) and (4) shows that in an elastic medium, the velocities of longitudinal and transverse waves depend only on the material parameters λ,μ,ρ.

### 2.2. Mechanical Impedance

The transmission of stress waves within a structure is influenced by the mechanical impedance $Z_{m}$ [27]. Mechanical impedance is a key parameter characterizing the dynamic properties of a structure. It is defined as the ratio of the harmonic force F(ω) applied at a point on the system to the resulting velocity response v(ω) at that point:

(5) $$Z_{m}\left(\omega \right)=\frac{F\left(\omega \right)}{v\left(\omega \right)}=c+j\omega m+\frac{k}{j\omega }$$

When a stress wave propagates through a structure and encounters an interface with different mechanical impedance, reflection and transmission occur. Considering a one-dimensional case where a wave travels from a medium with impedance $Z_{1}$ to a medium with impedance $Z_{2}$, the force reflection coefficient $R_{F}$ and force transmission coefficient $T_{F}$ can be expressed as follows:

(6) $$R_{F}=\frac{Z_{2}-Z_{1}}{Z_{1}+Z_{2}}$$

(7) $$T_{F}=\frac{2Z_{2}}{Z_{1}+Z_{2}}$$

In a bolted flange connection structure, when the bolt preload decreases, the compressive stress magnitude and degree of compression change. This causes the shear modulus of the gasket itself to change, affecting wave propagation within the gasket. Simultaneously, the contact stress between the flange face and the gasket surface is reduced, decreasing the compaction level. The impedance at the flange–gasket interface changes accordingly, altering the reflection and transmission coefficients at this interface, which in turn affects the propagation of stress waves [28].

In practical application scenarios, the gasket is usually in a pre-compressed state. According to incremental elasticity theory, the relationship between the wave velocity under pre-strain and the wave velocity under the initial state is as follows:

(8) $$\frac{c\left(\varepsilon_{0}\right)}{c_{0}}=1+\frac{1}{2}\left(\frac{C_{3}}{E}\right)\varepsilon_{0}$$

where $\varepsilon_{0}$ represents the pre-strain, and $C_{3}$ represents the third-order elastic constant. Research [29] indicates that the wave velocity of stress waves within a material is influenced by the magnitude of the pre-strain. In a pipe flange, when a bolt loosens, the tightening force in the bolted connection area decreases. Consequently, the pressure on the gasket is reduced, and the amount of pre-strain diminishes, which in turn affects the wave velocity of the stress wave within the gasket at that moment.

### 2.3. Finite Element Analysis of Bolt Loosening in Pipe Flanges

**Figure 1:**
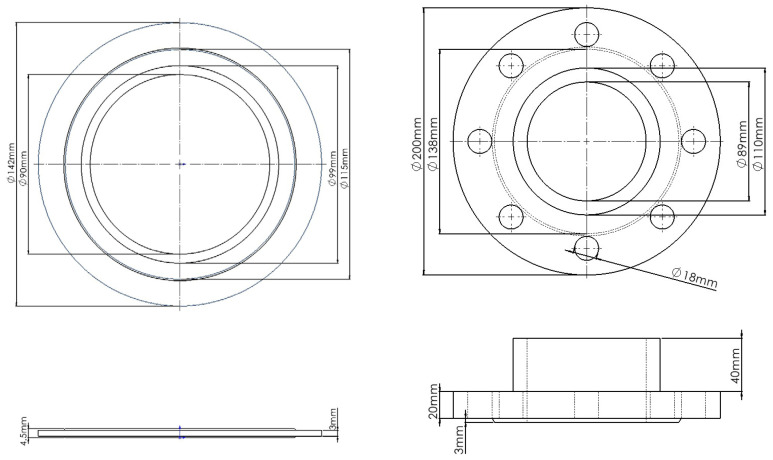
Flange and gasket sketch.

A model of the bolted flange connection sealing system was established. The flange under investigation is a PN40, DN80 integral flange, utilizing eight M16 bolts. The sealing gasket is a metallic spiral wound gasket. The specific dimensions of the flange disc and the gasket are illustrated in the Figure 1.

**Figure 2:**
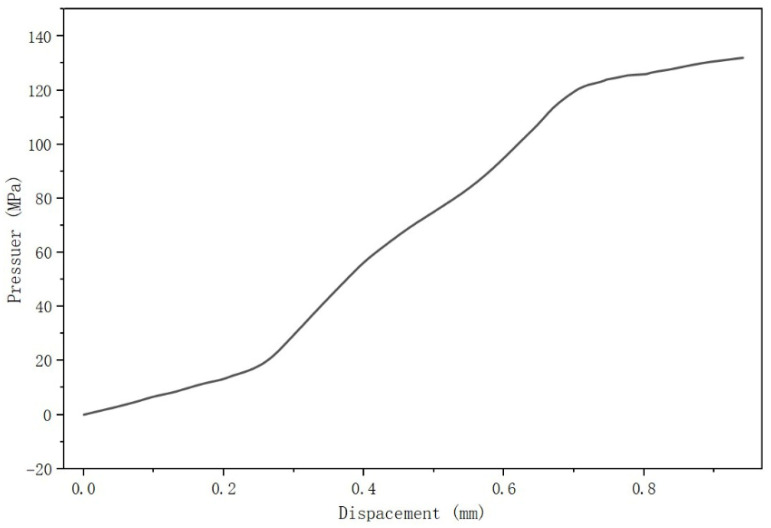
Gasket Pressure–Deformation Curve.

Based on the above parameters, the flange structure was modeled using Solid Works. The material for both the flanges and the bolts is 304 stainless steel. The filler material for the sealing gasket is flexible graphite, and the material for the gasket’s positioning ring is 304 stainless steel. The material properties for the flanges, bolts, and positioning ring were set as follows: density 7.93 g/cm^3^, Young’s modulus 194 GPa, and Poisson’s ratio 0.3. The gasket filler material was modeled using a gasket model, and the pressure–deformation curve provided by the gasket manufacturer is shown in the Figure 2.

**Figure 3:**
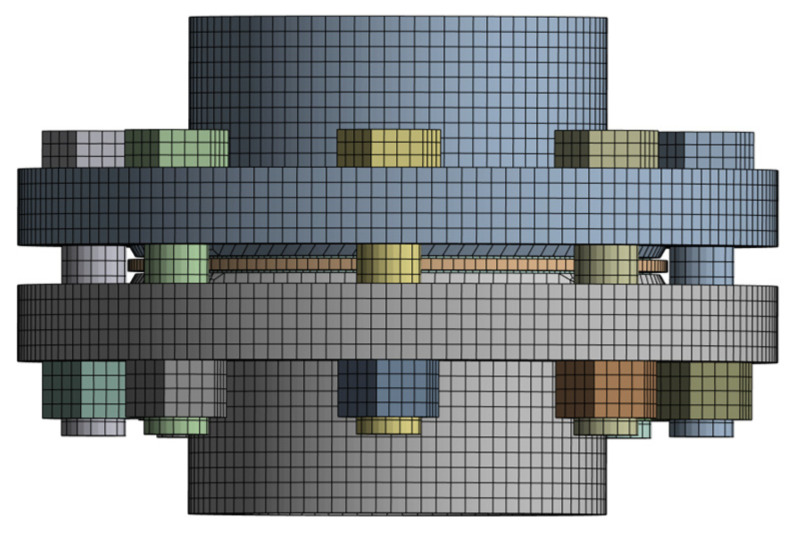
Mesh division.

Contact settings and meshing for the finite element model were performed in Workbench. The contact settings for the flange structure are shown in Table 1. The mesh size for the upper/lower flange discs, bolts, and nuts was 4 mm, with a tetrahedral-dominant method. The mesh size for the positioning ring of the sealing gasket was 3 mm, with a hexahedral-dominant method. The mesh for the filler part of the sealing gasket was a gasket-specific mesh, using a swept method with a mesh size of 2 mm, hexahedral-dominant. The finite element model of the flange structure is shown in Figure 3.

To simulate subsequent experiments, a fixed constraint was applied to the end face of the lower flange pipe.

According to GBT 150.3-2024 [30], the gasket compression force *F_c_* for a PN40 stainless steel spiral wound gasket is divided into the pre-tightening force $F_{a}$ and the operating force $F_{p}$. The calculation methods are shown in Formulas (9) and (10).

(9) $$F_{a}=\pi D_{G}by$$

(10) $$F_{p}=2b\pi D_{G}mp_{c}$$

In these formulas, $D_{G}$ represents the diameter of the gasket load reaction line, taken as the pitch circle diameter of the gasket. y represents the gasket seating stress, determined from tables as 69 MPa for this gasket. m represents the gasket factor, determined from tables as 3 for this gasket. $p_{c}$ represents the calculation pressure, taken as 4 MPa. b represents the effective gasket sealing width, the value of which is determined by the basic gasket seating width $b_{0}$. The calculated basic gasket seating width for this gasket is 8 mm. The formula for calculating the effective gasket sealing width is shown in Formula (11).

(11) $$b=2.53\sqrt{b_{0}}$$

Solving Formula (11) yields b = 7.156 mm. Substituting the above values into Formulas (9) and (10) gives $F_{a}$ = 173,735.00 N and $F_{p}$ = 60,429.57 N. The minimum required bolt load under pre-tightening and operating conditions is then calculated using Formulas (12) and (13).

(12) $$W_{a}=F_{a}$$

(13) $$W_{p}=0.785D_{G}^{2}p_{c}+F_{p}$$

The results obtained are $W_{a}$ = 173,735.00 N and $W_{p}$ = 99,817.73 N. Since $W_{a}$ > $W_{p}$, $W_{a}$ is taken as the bolt load for the flange. Consequently, the preload acting on each bolt is 21,716.88 N, rounded to 22,000 N as the preload for the flange bolts.

**Figure 4:**
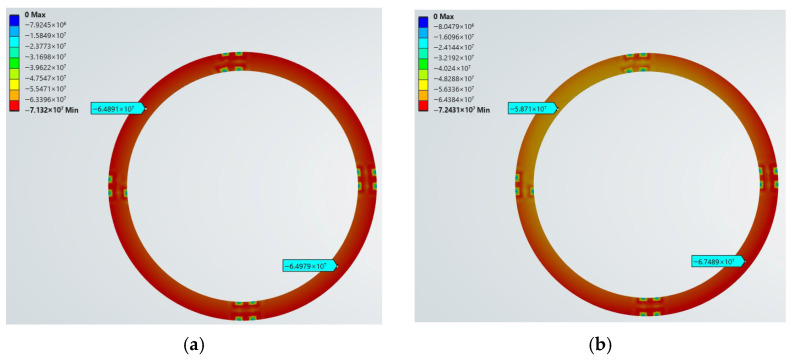

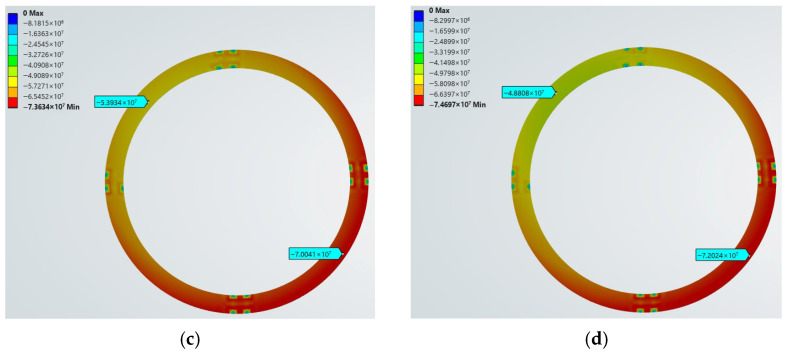
Gasket normal pressure. (**a**) 22,000 N (**b**) 18,333 N (**c**) 14,667 N (**d**) 11,000 N.

To investigate the influence of reduced bolt preload on the elastic deformation of the gasket, Workbench was used to perform simulation analyses of the gasket’s normal pressure under different preload levels. First, a preload of 22,000 N was applied to all bolts using the bolt preload function, and a simulation analysis was conducted. Subsequently, the preload on one specific bolt was set to 18,333 N (a reduction of approximately 1/6), while keeping the preloads on the other bolts unchanged, and another simulation analysis was performed. Following this gradient, simulations were completed for cases where the loose bolt had preloads of 14,667 N and 11,000 N. The contour plots of the gasket’s normal pressure distribution under different preloads are shown in Figure 4.

According to the normal pressure contour plots of the gasket, when a bolt loosens, in addition to the decrease in normal pressure on the gasket in the region of that loose bolt, the normal pressure on the gasket in the opposite region relatively increases. The overall pressure distribution on the gasket tends to become irregular. The slopes of the pressure-deformation curves for the gasket in the loose bolt region and the opposite tightened region under the above loosening conditions were calculated and regarded as the instantaneous elastic properties of the gasket at those times. Under the four bolt loosening conditions, the instantaneous elastic properties of the gasket in the loose bolt region and the opposite tightened region are shown in Table 2.

Based on the finite element simulation results, in a bolted flange connection structure, when the bolt preload decreases, the magnitude of compressive stress, the degree of compression, and the pressure distribution change. This leads to a change in the elastic modulus of the gasket itself, affecting the propagation performance of waves within the gasket. Simultaneously, the contact stress between the flange face and the gasket surface decreases, reducing the degree of compaction. The impedance at the flange–gasket interface changes accordingly, thus altering the reflection and transmission coefficients at this interface, which in turn affects the degree of stress wave attenuation within the flange structure. In response to the above analysis, this paper takes the flange structure containing a gasket as the research object, uses the hammer impact method to excite the structure, and collects vibration signals before and after bolt loosening to conduct research on bolt loosening detection in sealed pipe flanges.

## 3. Experiments and Discussions

### 3.1. Experiment Preparation

**Figure 5:**
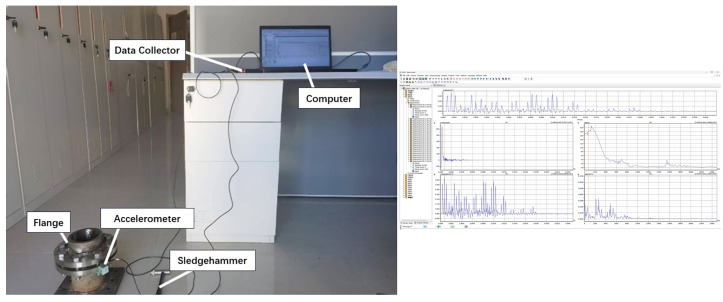
Test Bench.

A bolted flange connection sealing structure was established, as shown in Figure 5. The flange is a PN40, DN80 integral flange, utilizing eight M16 bolts and a metallic gasket. The sealing gasket is a spiral wound gasket, with both the flange and bolt materials being 304 stainless steel. The filler material of the gasket is flexible graphite, and its centering ring is also made of 304 stainless steel.

**Figure 6:**
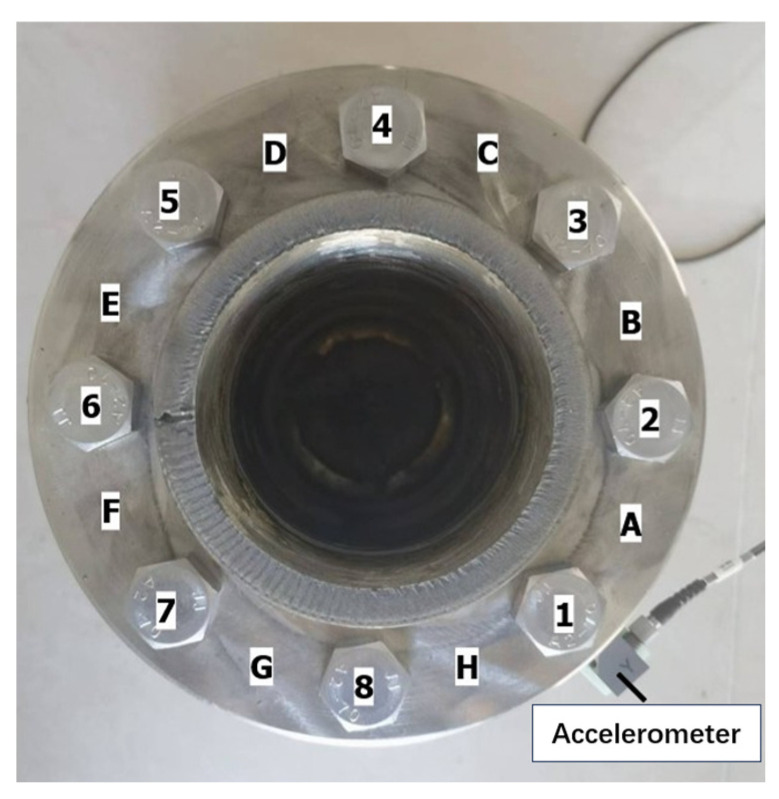
Schematic diagram of bolt positions and impact points.

Based on the thin plate bending vibration theory, the outer edge region of the flange structure exhibits the maximum displacement amplitude under the bending vibration mode, making it the most sensitive to bending vibrations. In this experiment, the acceleration sensor was attached to the outer edge of the lower flange side to ensure maximum acquisition of the flange bending vibration signals. Moreover, this placement guarantees that after excitation is applied on the upper flange surface, the sensor can capture the vibration signals that have propagated through the gasket. The selection of sensor installation location influences the modal components and amplitude of the acquired vibration signals; therefore, the sensor position was kept consistent throughout all tests in the experiment. Eight M16 bolts made of 304 stainless steel with strength grade A2-70 were evenly distributed on the flange. The eight bolts were sequentially numbered as Samples 1 to 8, and eight impact points, labeled A to H, were marked on the flange. The impact testing system is shown in Figure 5, and the specific bolt numbering along with impact positions is detailed in Figure 6.

The accelerometer selected for this study is the INV9836B-50 three-axis general-purpose piezoelectric accelerometer produced by Beijing Dongfang (Beijing, China), with a sensitivity of 100 mV/g and a range of ±50 g. The selected data acquisition device is the SA1802A data acquisition device produced by Wuxi Youtian Technology Co., Ltd. (Wuxi, China). This device’s signal input type is voltage, the signal input range is ±10 V, and the acquisition accuracy is better than 0.5%. The data acquisition and analysis system used is the SA1800 dynamic signal acquisition and analysis system produced by Wuxi Shiao Technology Co., Ltd. (Wuxi, China).

Based on the previous bolt preload calculation, the required preload for a single bolt is 22,000 N. According to GB/T 16823.2-1997 [31], the torque calculation formula is

(14) T=F·d·K

In this formula, T is the tightening torque, F is the bolt preload (taken as 22 kN), d is the nominal bolt diameter (16 mm), and K is the torque coefficient (taken as 0.2). The calculated tightening torque is 70.4 Nm. For convenience in subsequent tests, a preload corresponding to a torque of 75 Nm was calibrated as the initial state. This experiment utilized a torque wrench to calibrate the bolt tightening torque. The torque wrench model employed was the JWZ4-135BN manufactured by Shanghai Engel Mechanical & Electrical Equipment Co., Ltd. (Shanghai, China), which has a measurement range of 6.8 to 135 Nm and a torque accuracy of ±3%. This torque wrench is capable of applying a fixed torque to the bolts, thereby ensuring that the bolt tightening torque corresponds precisely to the value required for the experiment.

### 3.2. Bolt Loosening Detection Experiment

#### 3.2.1. Determination of Impact Force

**Figure 7:**
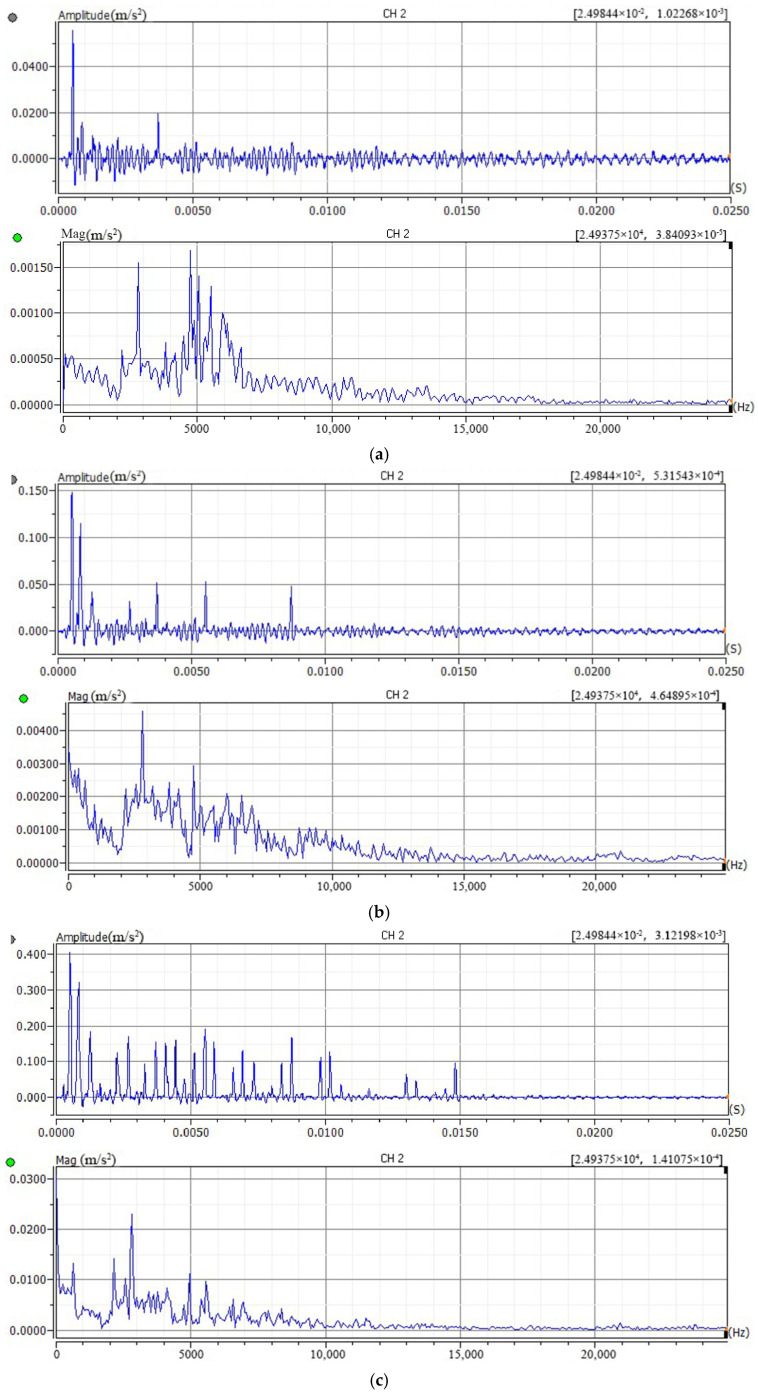

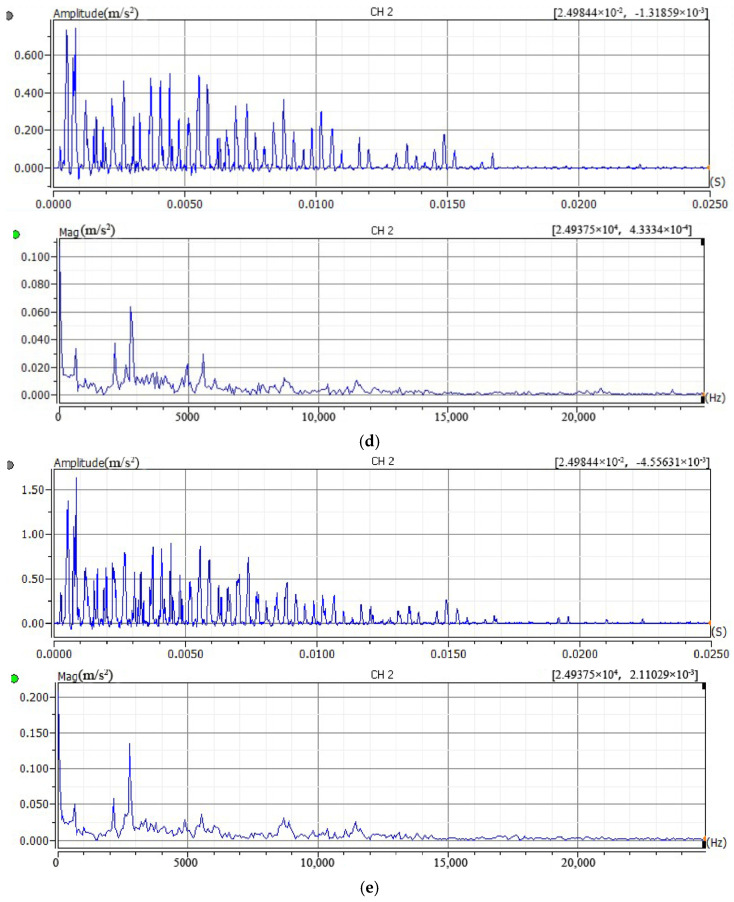
Signal time-domain and frequency-domain plots excited by different impact forces. (**a**) 300 N (**b**) 500 N (**c**) 700 N (**d**) 1000 N (**e**) 1500 N.

To determine the optimal impact force for the bolt loosening detection test, acceleration signals were measured under different impact forces and with different hammer head materials. First, all bolts were tightened to 75 Nm and allowed to rest for 5 min to eliminate short-term relaxation effects. Using a stainless steel hammer head, impact forces of approximately 300 N, 500 N, 700 N, 1000 N, and 1500 N were applied at point A on the upper flange surface. The time-domain and frequency-domain plots of the collected acceleration signals are shown in Figure 7.

Analysis of the results indicates that at impact forces of 300 N, 500 N, and 700 N, the characteristics of the collected vibration signals are incomplete. As the impact force increases, the number of wave peaks in the vibration signal waveform increases, and the waveform gradually stabilizes. New peaks appear in the frequency spectrum. When the impact force reaches 1000 N or more, the waveform and frequency spectrum show little change except for the peak magnitudes. To avoid potential overload of the impact hammer due to excessive force, 1000 N was selected as the optimal impact force.

#### 3.2.2. Determination of Hammer Head Material

**Figure 8:**
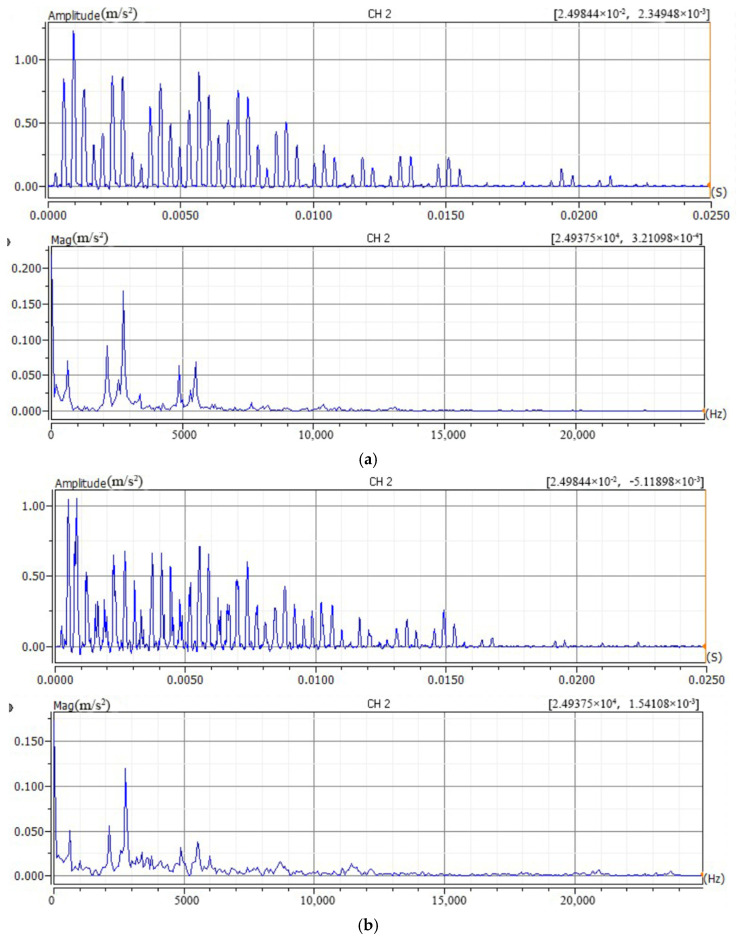

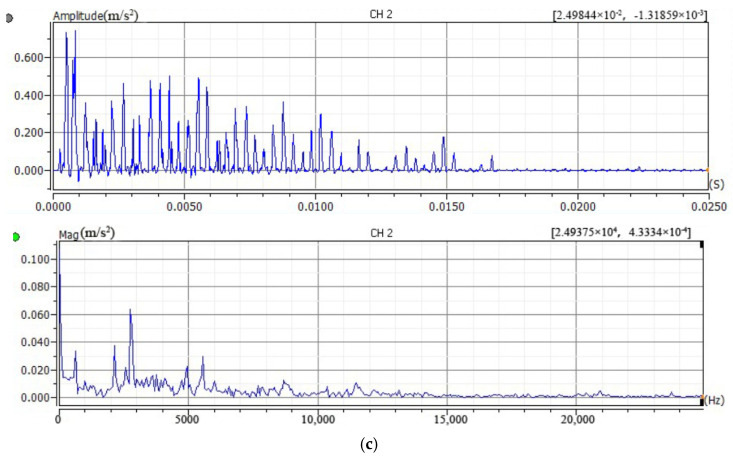
Signal time-domain and frequency-domain plots for different hammer materials under fully tightened condition. (**a**) Nylon hammer (**b**) Aluminum alloy hammer (**c**) Stainless steel hammer.

**Figure 9:**
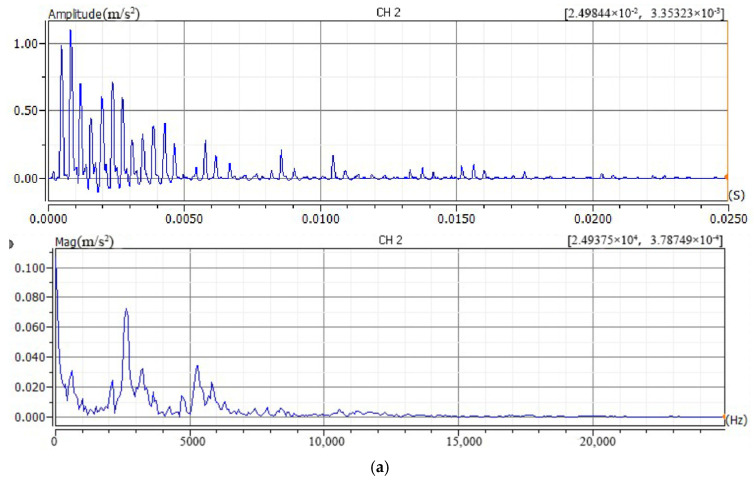

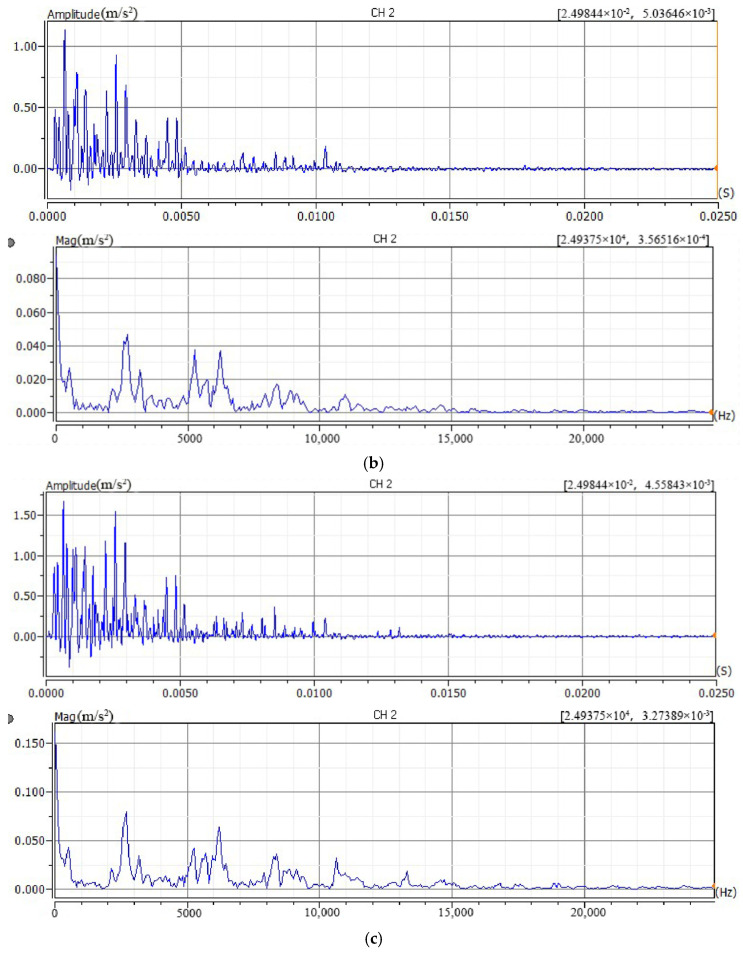
Signal time-domain and frequency-domain plots for different hammer materials when Bolt 2 is loose. (**a**) Nylon hammer (**b**) Aluminum alloy hammer (**c**) Stainless steel hammer.

To determine the optimal hammer head material for the bolt loosening detection test, all bolts were initially tightened to 75 Nm. Hammer heads made of nylon, aluminum alloy, and stainless steel were used to strike point A on the upper flange with an impact force of 1000 N. After striking, all bolts were loosened. Subsequently, all bolts except Bolt 2 were tightened to 75 Nm. Bolt 2 was tightened to 62.5 Nm. After tightening, the setup rested for 5 min. Then, the three hammer head types were used again to strike point A on the upper flange with 1000 N force. The collected time-domain and frequency-domain plots of the acceleration signals are shown in Figure 8 and Figure 9.

Analysis of the time-domain graphs indicates that the waveforms generated by the three hammer materials do not show significant differences overall. The signal waveform produced by the nylon hammer head appears relatively smooth. Frequency spectrum analysis shows that the signal frequency excited by the nylon hammer head can reach 6000 Hz; the signal frequency excited by the aluminum alloy hammer head can reach 12,000 Hz; and the signal frequency excited by the stainless steel hammer head can reach 15,000 Hz. Among them, the signal excited by the nylon hammer head has the lowest noise, and the significant frequency changes caused by bolt loosening are mainly concentrated in the 2000–12,000 Hz range, which coincides with the excitation frequency range of the aluminum alloy and stainless steel hammer heads. To avoid missing features in the 6000–12,000 Hz frequency band caused by bolt loosening, an aluminum alloy hammer head, which can excite frequencies up to 12,000 Hz and has relatively low metal noise, is used for subsequent impact tests.

#### 3.2.3. Test Result Analysis

To investigate the capability of the adopted method for detecting the degree of bolt loosening, all bolts were initially torqued to 75 Nm and allowed to rest for 5 min to eliminate short-term relaxation effects. The sampling frequency of the data acquisition system was configured to 64,000 Hz. An aluminum alloy hammer head was installed on the impact hammer. Using an impact force of 1000 N at point C on the upper flange surface, the acceleration signal generated by the impact was recorded to establish the baseline condition with all bolts fully tightened.

Subsequently, Bolt 2 was completely loosened and then retightened to a torque of 62.5 Nm. After another 5 min rest period, the flange was struck with the same force (1000 N) at point C, and the signal was recorded. Following this, the preload on Bolt 2 was progressively reduced in increments of 12.5 Nm, down to a final torque of 37.5 Nm. Measurements were taken and results saved after each incremental loosening step.

**Figure 10:**
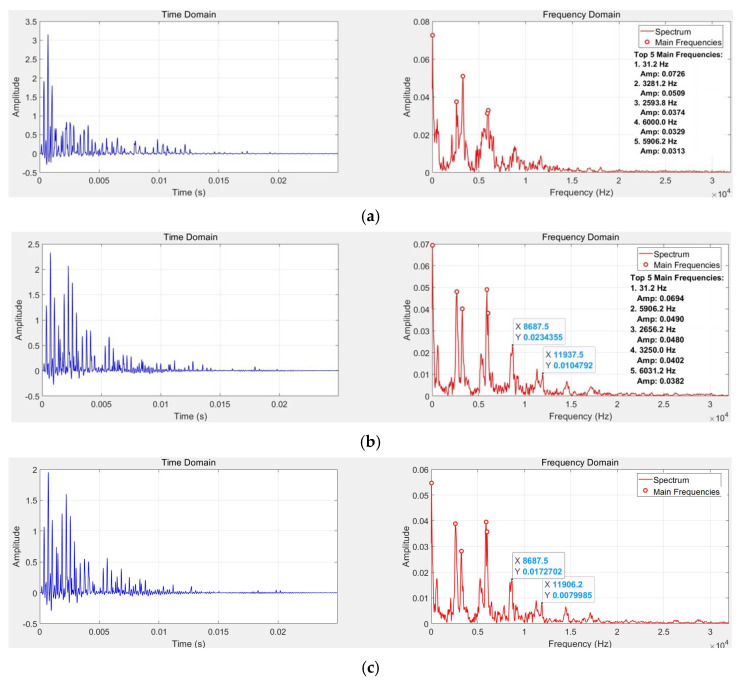

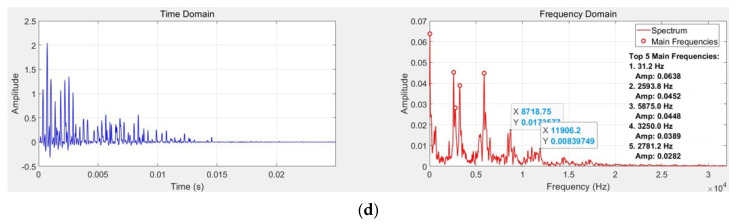
Signal time−domain and frequency−domain plots for Bolt 2 under different loosening conditions. (**a**) 75 Nm (**b**) 62.5 Nm (**c**) 50 Nm (**d**) 37.5 Nm.

The acceleration data acquired by the sensor were plotted and analyzed to assess the accuracy and repeatability of the experimental outcomes. Based on the collected data, curves were generated for the fully tightened condition and for conditions where individual bolts (1 through 8) were loosened. Figure 10 presents the time-domain and frequency-domain plots of the signals for Bolt 2 under various tightening torque conditions ranging from 75 Nm to 37.5 Nm.

Analysis of the signal processing results reveals that as the bolt tightening torque decreases to 62.5 Nm, the spectral structure of the signal undergoes significant changes. Specifically, the peak amplitudes at approximately 6000 Hz and 5906 Hz increase markedly, while the peak at 3250 Hz diminishes, and a peak near 2656 Hz becomes more prominent. In addition, changes in the spectral structure around 8000 Hz and 12,000 Hz can be observed. When the tightening torque decays to 50 Nm, the overall spectral structure does not exhibit substantial alterations compared to the 62.5 Nm state; however, the amplitudes of the dominant frequency components show a general decrease. Upon further preload reduction to 37.5 Nm, the peak around 6000 Hz disappears entirely, a new peak emerges at approximately 2781 Hz, and the amplitudes of several main frequency components recover to some extent.

**Figure 11:**
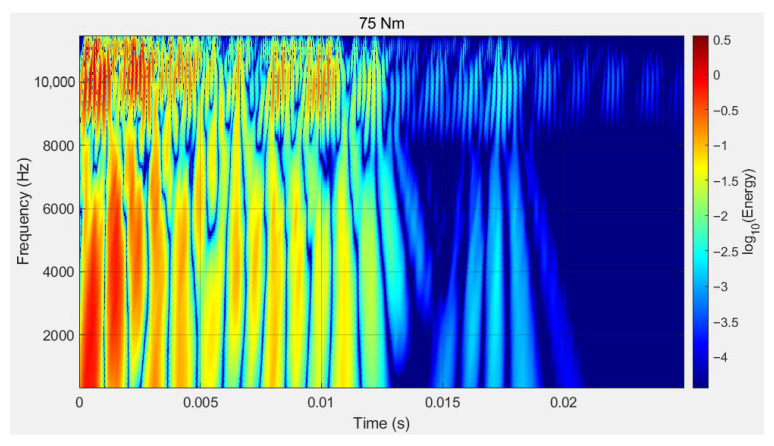

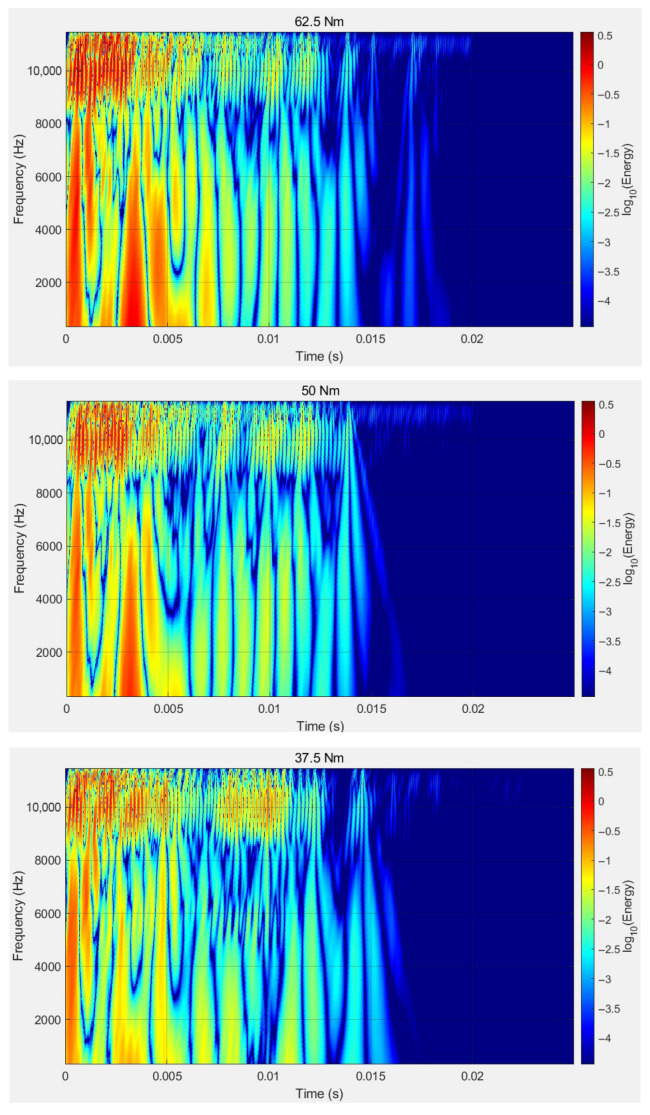
Wavelet scalogram of vibration signals under different looseness conditions.

According to the study in 3.2.2 and the spectral analysis under four bolt loosening conditions, it can be seen that after the bolts loosen, the changes in the signal spectrum structure are mainly concentrated in the 0–12,000 Hz range. To reduce computational analysis costs, the subsequent frequency domain analysis of the signals in this paper will mainly focus on the 0–12,000 Hz range. Figure 11 shows the wavelet scalogram of the signal in the 0–12,000 Hz range.

Based on Figure 11, when the bolt is fully tightened, the signal energy is mainly concentrated around 2000 Hz and 4000 Hz, and the time-frequency structure appears regular and compact. This indicates that under fully tightened conditions, the flange–gasket interface maintains good contact, resulting in a relatively regular dynamic response of the structure. When the bolt is loosened to 62.5 Nm, the overall signal energy decreases, but energy disperses to around 8000 Hz in the initial time domain. The energy within the 2000–6000 Hz range becomes comparable, and the time-domain attenuation pattern shows some degree of irregularity. Between 0.005 s and 0.01 s, the energy near 8000 Hz is extremely low, while the high-frequency energy near 10,000 Hz decays more slowly. When the preload is further reduced to 50 Nm, the overall signal energy continues to decline, but the change in frequency-domain energy distribution is relatively small. Energy decay accelerates, and near 0.005 s, the energy around 4000 Hz and 8000 Hz becomes extremely low. The irregularity of the time-domain attenuation pattern becomes more severe. When the bolt is loosened to 37.5 Nm, between 0.005 s and 0.01 s, the stripes in the 0–5000 Hz range become wider, while those in the 6000–8000 Hz range become narrower and more numerous. This indicates a change in the time-domain energy distribution, reflecting significant variations in both time and frequency domains under severe bolt loosening conditions. By comparing and analyzing the spectral composition of the signals as described above, different levels of bolt tightening torque can be distinguished. This demonstrates the feasibility of the adopted method for identifying the degree of bolt loosening.

## 4. Detection Model and Optimization

### 4.1. Multi-Domain Feature Extraction and Comparative Analysis of Bolt Loosening Recognition Models

#### 4.1.1. Construction of Multi-Domain Feature System

Vibration signals contain rich structural state information, and features from a single domain often prove insufficient to comprehensively characterize the dynamic behavioral changes induced by bolt loosening. To fully exploit the feature information sensitive to bolt loosening within vibration signals, this study constructs a multi-domain feature system encompassing four dimensions: time domain, frequency domain, time-frequency domain, and nonlinear dynamics, thereby achieving refined characterization of bolt loosening states [28,29].

Time-domain statistical features are important methods for analyzing time-series signals and are widely used in fields such as mechanical fault diagnosis and signal processing. By extracting time-domain features, the fluctuation, energy distribution, and morphological characteristics of a signal can be effectively described. This paper extracts five time-domain features: root mean square (RMS), kurtosis, crest factor, shape factor, and impulse factor.

The root mean square (RMS) represents the equivalent DC value of the average energy or power of a signal or function over one cycle.

(15) $$\begin{array}{c} RMS=\sqrt{\sum_{n=1}^{n}x{\left[n\right]}^{2}/N} \end{array}$$

Kurtosis is a statistical measure that describes the peakedness of a probability distribution. It quantifies the thickness of the distribution’s tails and the concentration of data near the mean. In fault diagnosis, kurtosis is highly sensitive to impulsive transients—when sudden impacts occur in a signal, the kurtosis value increases significantly.

(16) $$K=\frac{n\left(n+1\right)}{\left(n-1\right)\left(n-2\right)\left(n-3\right)}\sum_{i=1}^{n}{\left(\frac{x_{i}-\bar{x}}{s}\right)}^{4}-\frac{3{\left(n-1\right)}^{2}}{\left(n-2\right)\left(n-3\right)}$$

where n is the sample size, $x_{i}$ is the i-th data value, $\bar{x}$ is the sample mean, and s is the sample standard deviation.

The crest factor is the ratio of the signal’s peak value to its RMS value, reflecting the prominence of instantaneous peaks relative to the average energy. For normal vibration signals without impacts, the crest factor is typically small; when periodic impacts occur, the crest factor increases significantly.

(17) $$CF=\frac{\max\left|x\left(n\right)\right|}{X_{RMS}}$$

The shape factor is the ratio of the RMS value to the rectified mean value, used to describe the overall waveform shape. The rectified mean is the average of the absolute values of the signal. In mechanical fault diagnosis, the shape factor can help distinguish waveform changes caused by different fault types, such as wear or looseness.

(18) $$SF=\frac{X_{RMS}}{\frac{1}{N}\sum_{n=1}^{N}\left|x\left(n\right)\right|}$$

The impulse factor is the ratio of the signal’s peak value to its rectified mean value. Similarly to the crest factor, the impulse factor uses the rectified mean as the denominator and is more sensitive to transient pulses with large amplitudes. It is often used to detect discrete impulsive components in a signal.

(19) $$IF=\frac{\max\left|x\left(n\right)\right|}{\frac{1}{N}\sum_{n=1}^{N}\left|x\left(n\right)\right|}$$

Frequency-domain features are parametric quantities extracted after converting a signal from the time domain to the frequency domain using tools such as the Fourier transform. By analyzing the distribution and energy intensity of different frequency components within a signal, these features reveal periodic patterns, harmonic structures, and energy concentration zones that are difficult to observe directly in the time dimension. Common frequency-domain features include the spectral centroid, frequency variance, spectral entropy, and band energy. These features can effectively characterize the vibration modes, state characteristics, and fault information of a signal. This paper extracts the spectral centroid and spectral entropy of the signal.

The spectral centroid represents the center of gravity of the energy distribution in the frequency domain, reflecting the central frequency of the signal’s spectrum. It is obtained by weighting each frequency component by its amplitude. In mechanical fault diagnosis, when equipment experiences wear, looseness, or impact faults, the spectral centroid shifts. For a discrete spectrum with frequency points $f_{k}$ and corresponding amplitude spectra $A_{k}$ (k = 1, 2, …, K), the spectral centroid is

(20) $$SC=\frac{\sum_{k=1}^{K}f_{k}\cdot A_{k}}{\sum_{k=1}^{K}A_{k}}$$

Spectral entropy measures the uncertainty or complexity of a signal’s spectrum. When a system is fault-free, the spectral energy is concentrated in a few frequencies, resulting in low spectral entropy. When the signal contains complex components, noise, or multi-frequency modulation caused by faults, the spectral distribution becomes more uniform, and the spectral entropy increases. To calculate spectral entropy, the power spectrum *P_k_* is first normalized to a probability distribution.

(21) $$p_{k}=\frac{P_{k}}{\sum_{j=1}^{K}P_{j}}$$

Then, the spectral entropy is defined as

(22) $$SE=-\sum_{k=1}^{K}p_{k}\cdot \log_{2}\left(p_{k}\right)$$

Time-frequency domain features are proposed for analyzing non-stationary signals. By describing the local characteristics of a signal in the joint time-frequency dimension, it is revealed how signal energy, frequency, and other features evolve over time. Unlike frequency-domain analysis, which only displays global frequency distribution, time-frequency analysis excels at capturing transient phenomena, abrupt changes, and dynamic frequency shifts. Its core lies in visually presenting signal components on a two-dimensional time-frequency plane through a time-frequency distribution function. This paper extracts the wavelet packet band energy ratio and wavelet packet band entropy of the signal.

When bolt loosening occurs, the energy of the vibration signal redistributes across different frequency bands. Wavelet packet decomposition enables refined decomposition of both the high-frequency and low-frequency components of a signal, making it particularly suitable for analyzing non-stationary vibration signals [28,29]. After preprocessing, the signal undergoes an n-level wavelet packet decomposition, yielding 2^n^ equal-width frequency band components. The normalized energy ratio of each band forms a feature vector. This feature sensitively reflects the energy redistribution across bands caused by changes in contact conditions [32]. In this study, the db4 wavelet basis is selected for 4-level wavelet packet decomposition, producing 16 equal-width frequency bands. The db4 wavelet basis offers strong time-domain localization, non-redundant decomposition, and constant energy characteristics. For a discrete signal x(t), let the coefficient at the j-th level and n-th node be $d_{j}^{n}\left(k\right)$:

(23) $$d_{j+1}^{2n}\left(k\right)=\sum h\left(m-2k\right)d_{j}^{n}\left(m\right)$$

(24) $$d_{j+1}^{2n+1}\left(k\right)=\sum_{m}g\left(m-2k\right)d_{j}^{n}\left(m\right)$$

where $d_{0}^{0}\left(k\right)=x\left(k\right)$ is the original signal, j = 0, 1, 2, …, J (J is the number of decomposition levels), h(k) is the low-pass filter coefficient, g(k) is the high-pass filter coefficient, and m−2k represents the shift of the filter coefficient relative to the input sequence.

Assume an n-level wavelet packet decomposition of the signal. The decomposition coefficient of the i-th frequency band is $d_{i}(k)$, with $k=1,2,\ldots ,L_{i}$, where $L_{i}$ is the length of the coefficient of the i-th band. The energy of the i-th band is as follows:

(25) $$E_{i}=\sum_{k=1}^{L_{i}}{\left|d_{i}\left(k\right)\right|}^{2}$$

The normalized energy ratio of each frequency band is calculated as follows:

(26) $$p_{i}=\frac{E_{i}}{\sum_{j=0}^{2^{n}-1}E_{j}}$$

The normalized energy ratio satisfies the following formula:

(27) $$\sum_{i=0}^{2^{n}-1}p_{i}=1,0\leq p_{i}\leq 1$$

The resulting feature vector is as follows:

(28) $$F=\left[p_{0},p_{1},p_{2},\ldots ,p_{2^{n}-1}\right]$$

Based on the analysis in Section 3.2, the portion of the signal spectrum where the spectral structure undergoes significant changes after bolt loosening is concentrated within the range of 0–12,000 Hz. Therefore, this study primarily calculates the wavelet packet band energy ratio for the experimental signal data within this range. The formula for calculating the band width is as follows:

(29) $$\Delta f=F_{s}/2^{4}=12,000/16=750\;\mathrm{Hz}\;$$

The width of each frequency band is 750 Hz. Starting from 0, Band 1 covers the range of 0–750 Hz, Band 2 covers 750–1500 Hz, …, and Band 16 covers the range of 11,250–12,000 Hz.

Based on the wavelet packet band energy ratio, the wavelet packet band entropy is further defined to characterize changes in the energy distribution pattern as a whole [33]. When bolts are fully tightened, the structural dynamic characteristics are relatively stable, and energy is concentrated in a few frequency bands associated with the structural modes, resulting in low band entropy. When bolts loosen, the change in interface contact conditions causes energy to redistribute across bands, leading to a more uniform energy distribution and an increase in band entropy. Research indicates that wavelet packet entropy is highly sensitive to bolt loosening and can serve as an effective damage indicator [33]. Wavelet packet band entropy is calculated as follows:

(30) $$H=-\sum_{i=0}^{2^{n}-1}p_{i}\cdot \log_{2}\left(p_{i}\right)$$

#### 4.1.2. Comparative Analysis of Bolt Loosening Recognition Based on Multiple Models

To transcend the limitations of single models in bolt loosening recognition, this study introduces three machine learning models in conjunction with two feature selection methods, constructing a multi-model comparative analysis framework.

SVM maps input features into high-dimensional space via kernel functions to identify the optimal hyperplane for classification, demonstrating excellent generalization capability in small-sample learning scenarios [34]. This study employs the Radial Basis Function (RBF) as the kernel function. The core advantages of SVM include a solid theoretical foundation (statistical learning theory, VC dimension theory), capability to handle nonlinear problems through kernel tricks, effectiveness with high-dimensional data, and favorable generalization under small-sample conditions. In the domain of bolt loosening detection, SVM has been extensively applied with promising results. In reference [34], SVM based on acoustic signatures achieved 89.93% accuracy in bolt loosening detection for transmission towers.

Random Forest is an ensemble learning algorithm based on the Bagging strategy, which constructs multiple decision trees and employs majority voting for classification [35]. This algorithm offers advantages including strong resistance to overfitting, capability to evaluate feature importance, and adaptability to nonlinear data. Random Forest performs exceptionally well in scenarios characterized by high dimensionality, nonlinearity, and complex relationships, and has been widely applied in fault classification tasks.

Bagging (bootstrap aggregating) involves sampling with replacement from the training set to generate multiple training subsets, training individual base learners, and integrating their prediction results. Compared to iterative ensemble methods such as AdaBoost, Bagging demonstrates greater stability under small-sample conditions without convergence failures. By reducing model variance, Bagging effectively enhances generalization capability [36].

The one-dimensional convolutional neural network (1D-CNN) is a special form of CNN, where the convolution kernel performs sliding convolution operations along a single time dimension, making it particularly suitable for processing one-dimensional time-series signals such as vibration and sound. Compared with two-dimensional CNN, 1D-CNN has the advantages of fewer parameters, higher computational efficiency, and end-to-end automatic feature extraction, eliminating the need for manually designed time-frequency features. In recent years, it has gained increasing attention in the field of bolt loosening detection. Hu et al. [37] proposed a 1D-CNN regression model combined with explainable artificial intelligence (XAI) (referred to as FS-X1D-CNN) for locating loose bolts and predicting torque in a 16-bolt connected aluminum plate. Under varying temperature conditions, the model achieved a localization accuracy of 5.95 mm and a torque prediction accuracy of 0.54 Nm, while converging 10 times faster than the original 1D-CNN.

RFE (recursive feature elimination) is a wrapper-type feature selection method based on model weights that recursively trains models and eliminates the least important features to obtain feature importance rankings [38]. For linear SVM, feature importance can be measured by the absolute values of the weight vector. Research demonstrates that RFE effectively reduces data dimensionality while retaining diagnostic information, enabling more accurate classification. In reference [38], the proposed RFE-assisted Transformer-SVM framework achieved a 15.72% improvement in accuracy compared to traditional SVM for multi-bolt connection loosening identification.

Lasso (least absolute shrinkage and selection operator) achieves feature coefficient sparsity by incorporating an L1 regularization term into the loss function, automatically completing feature selection. The L1 regularization compresses coefficients of unimportant features to zero, thereby identifying critical feature subsets. This method offers high computational efficiency and is suitable for high-dimensional feature scenarios.

#### 4.1.3. Dataset Preparation and Model Comparison

Following the experimental methodology described in Section 3.2.3, 20 vibration signals were collected at point C using 1000 N impact force under four tightening torque conditions for bolt No. 2: 75 Nm, 62.5 Nm, 50 Nm, and 37.5 Nm, yielding a total of 80 vibration signals.

One data sample was selected from each of the four loosening condition datasets, and the aforementioned feature analysis was performed. The results are presented in Table 3.

These 80 signals were randomly partitioned into training and test sets at a 7:3 ratio. Bolt loosening recognition was performed using randomized combinations of the aforementioned feature extraction methods and detection models. To mitigate the contingency of detection results, each model combination underwent 25 iterations of random dataset partitioning and detection, with mean accuracy and standard deviation calculated from the 25 detection results. The detection accuracies for each model combination are presented in Table 4.

According to the experimental results, all detection schemes achieved mean accuracies exceeding 90%. Among these, SVM-RFE demonstrated superior detection accuracy and stability compared to other schemes, with the shortest training time.

### 4.2. Result Analysis

**Figure 12:**
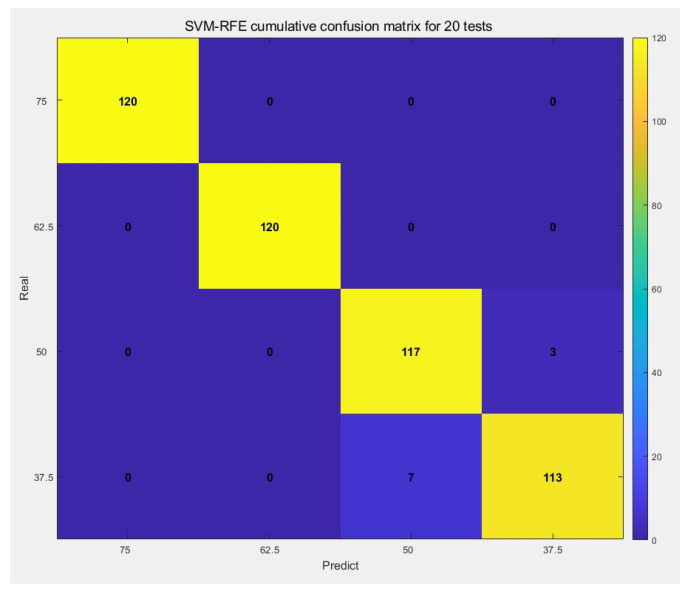
Overall Confusion Matrix of SVM-RFE.

**Figure 13:**
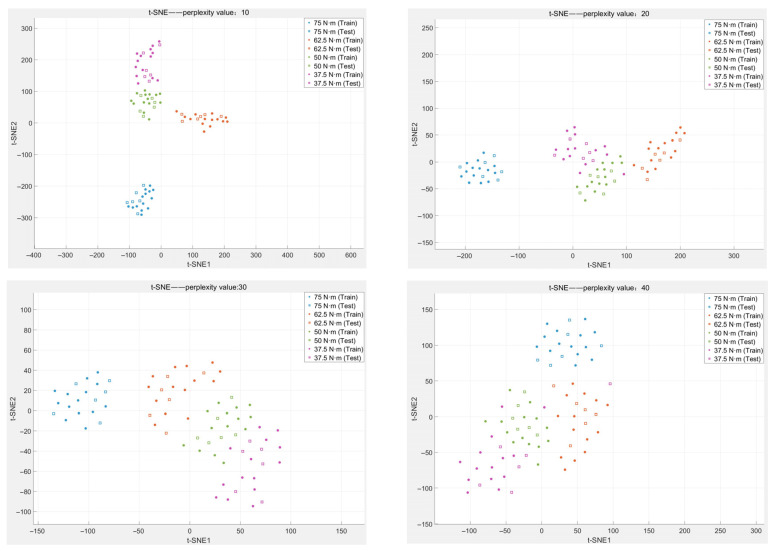
Sample Feature Distribution.

Figure 12 shows an overall confusion matrix after 20 SVM-RFE detections and Figure 13 shows the sample feature distribution under different perplexity values.

Based on the overall confusion matrix of 20 SVM-RFE detection iterations, this detection scheme exhibits excellent recognition capability for both complete bolt tightening and slight loosening. However, minor misclassifications occur under the 50 Nm and 37.5 Nm torque conditions, though overall recognition performance remains satisfactory. The sample feature distribution diagram reveals that under four different perplexity conditions, the vibration signal samples corresponding to varying tightening levels form relatively dispersed sample clusters. Notably, the sample cluster associated with the fully tightened condition is considerably distant from the clusters representing the three loosening conditions. This observation indicates that the adopted detection method possesses exceptionally high sensitivity to slight loosening of pipeline flange bolts and can accurately and efficiently achieve recognition of the degree of bolt loosening.

**Figure 14:**
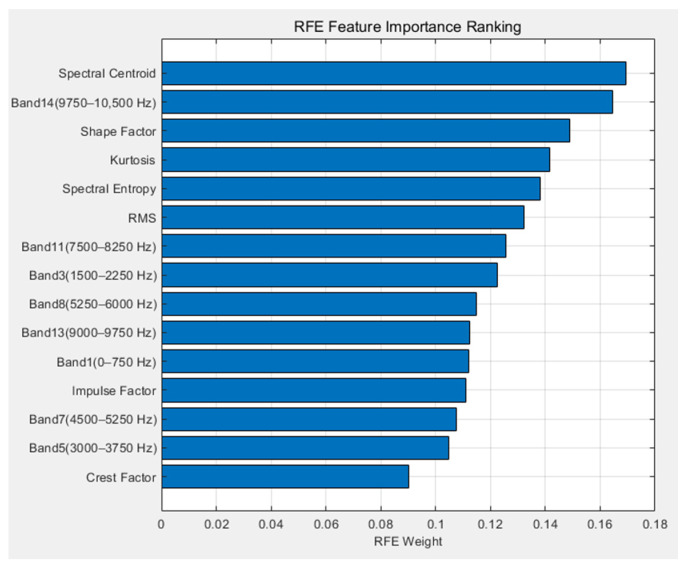
Feature Weight Ranking.

To investigate the influence of individual feature values on bolt loosening degree recognition, the feature weights employed in SVM-RFE detection were calculated, with results presented in Figure 14.

**Figure 15:**
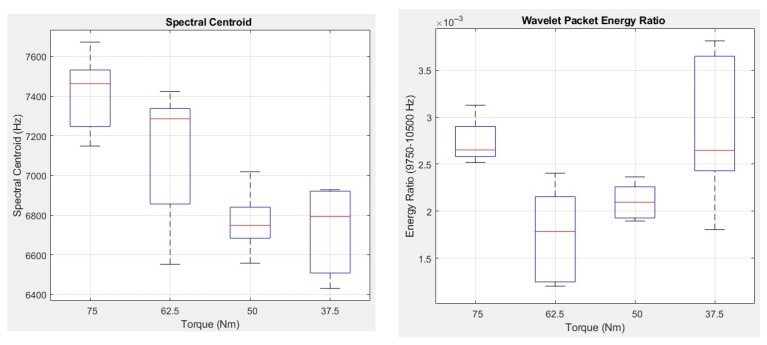

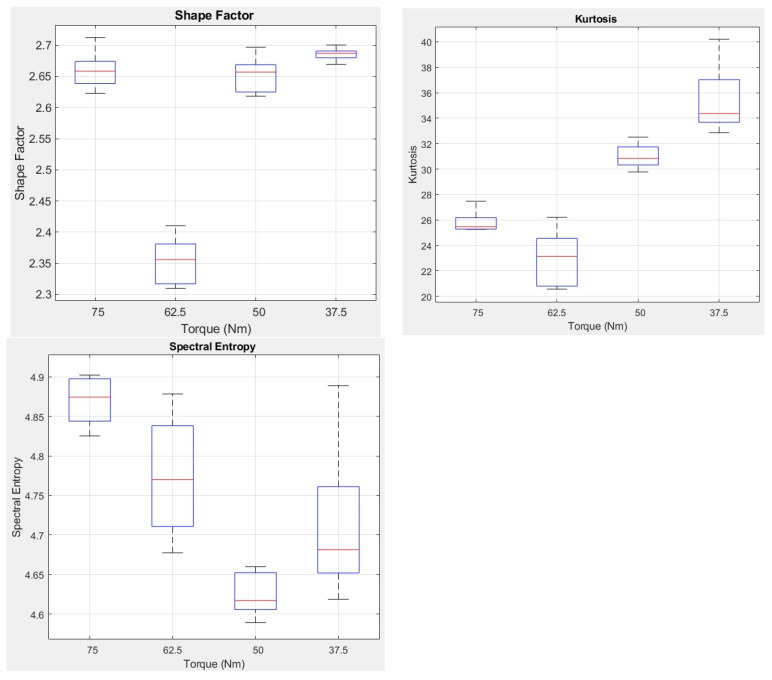
Feature Value Variation with Tightening Torque.

The figure indicates that the top five features influencing bolt loosening degree judgment in SVM-RFE are spectral centroid, wavelet packet band energy ratio (9750–10,500 Hz), shape factor, kurtosis, and spectral entropy. Twenty samples from each tightening gradient for calculation of these five feature values, with variations corresponding to changes in bolt tightening torque gradient, are illustrated in Figure 15.

From the analysis of the results, it can be observed that when bolt loosening occurs, the aforementioned five feature values exhibit significant changes. When the tightening torque of the bolt decreases from 62.5 Nm to 50 Nm, due to the intensification of the breathing effect and changes in gasket performance, each feature value undergoes a marked transformation. When the tightening torque further decreases from 50 Nm to 37.5 Nm, the change in the shape factor of the signal is minimal. This may be because, under these two loosening conditions, the breathing effect reaches saturation and the instantaneous elastic performance of the gasket exhibits relatively minor changes. Furthermore, it is observed that at a tightening torque of 37.5 Nm, apart from the shape factor, the distribution of each feature value across different signal samples is more dispersed compared to those under other torque gradients. The distribution of the spectral entropy feature value among samples is particularly scattered. According to the literature [39], under high preload, the entropy value exhibits a linear monotonic decreasing trend with torque, and the Pearson coefficient for linear fitting is greater than 0.99. However, in the low preload region, a stable pressure distribution has not yet formed on the contact surface, making the dynamic behavior of the system extremely sensitive to changes in boundary conditions. Due to minor differences in the knock point and knock force during each excitation, the nonlinear effects generated by each knock vary, leading to significant discrepancies in spectral entropy across multiple measurements. This results in a weaker identification capability of SVM-RFE for bolt loosening at a tightening torque of 37.5 Nm.

### 4.3. Optimization of Bolt Loosening Identification Method

To improve the accuracy of identifying bolt loosening signals under a tightening torque of 37.5 Nm, feature enhancement is performed based on the aforementioned SVM-RFE method, and a Cost-Sensitive Support Vector Machine model is introduced.

#### Feature Enhancement

To address the issue of significant variability among signal samples when the bolt tightening torque is 37.5 Nm, three feature values—Sample Entropy, Coefficient of Variation, and Higher-Order Statistics—were introduced. Sample Entropy is used to measure the complexity and regularity of time series data, and its formula is

(31) $$SampEn\left(m,r\right)=-\ln\left(\frac{A}{B}\right)$$

where B is the number of vector pairs that match in an m-dimensional space, and A is the number of vector pairs that match in an (m + 1)-dimensional space. A lower Sample Entropy value indicates higher self-similarity of the sequence, while a higher Sample Entropy value suggests a more complex and unpredictable sequence. Under low preload conditions, micro-slip at the contact interface leads to increased complexity in the vibration signals, and Sample Entropy can effectively capture this change [40].

The Coefficient of Variation measures the relative volatility of a set of data, i.e., the degree of dispersion relative to its mean. In the 37.5 Nm condition, due to the unstable pressure distribution on the contact surface, the response from each knock exhibits variability, causing significant fluctuations in the statistical quantities of each signal segment. This volatility itself becomes an effective indicator for identifying severe loosening.

Higher-order statistics describe the tail characteristics of the signal’s probability distribution, with the formula:

(32) $$M_{k}=E\left[{\left(X-\mu \right)}^{k}\right]/\sigma^{k}$$

Higher-order moments are extremely sensitive to transient shocks and outliers in the signal. Under severe loosening conditions, the nonlinear impacts caused by the breathing effect generate heavy-tailed distributions, and higher-order statistics can effectively capture these subtle but critical fault features [40].

### 4.4. Cost-Sensitive Support Vector Machine

Cost-Sensitive Support Vector Machine (CS-SVM) is an improvement upon the standard SVM. Its core idea is to introduce different penalty parameters for misclassified samples from different classes, aiming to minimize the total cost of misclassification [41]. The core of cost-sensitive learning is the cost matrix C, where C(i,j) represents the cost of misclassifying a sample from class i as class j. For a K-class classification problem, the general form of the cost matrix is

(33) $$C=\left[\begin{array}{ccccc} 0 & c_{12} & c_{13} & \cdots & c_{1K}\\ c_{21} & 0 & c_{23} & \cdots & c_{2K}\\ c_{31} & c_{32} & 0 & \cdots & c_{3K}\\ \vdots & \vdots & \vdots & \ddots & \vdots \\ c_{K1} & c_{K2} & c_{K3} & \cdots & 0 \end{array}\right]$$

In this matrix, the diagonal elements C(i,i)=0 indicate that the cost of correct classification is zero, while the off-diagonal elements C(i,j)>0 represent the cost of misclassifying a sample from class i as class j. The cost matrix configured for this experiment is as follows:

(34) $$C=\left[\begin{array}{cccc} 0 & 1 & 1 & 1.5\\ 1 & 0 & 1 & 1.5\\ 1 & 1 & 0 & 1.5\\ 3 & 3 & 3 & 0 \end{array}\right]$$

By setting the misclassification cost for the 37.5 Nm category to 3, the sample weight for this category is effectively increased threefold during the SVM training process. This strategy compels the classifier to reserve a larger decision region for the 37.5 Nm category within the feature space, thereby enabling it to effectively capture the dispersed sample distribution characteristic of this category.

Use the radial basis function (RBF) as the kernel function of SVM:

(35) $$K\left(x_{i},x_{j}\right)=\exp\left(-\gamma |x_{i}-x_{j}{|}^{2}\right)$$

The kernel width parameter γ is set as the reciprocal of the median distance between training samples. Box constraint parameter $C_{SVM}=1$. A one-versus-one error-correcting output coding strategy is adopted to decompose the four-class classification problem into six binary SVM subproblems.

### 4.5. Results and Analysis

**Figure 16:**
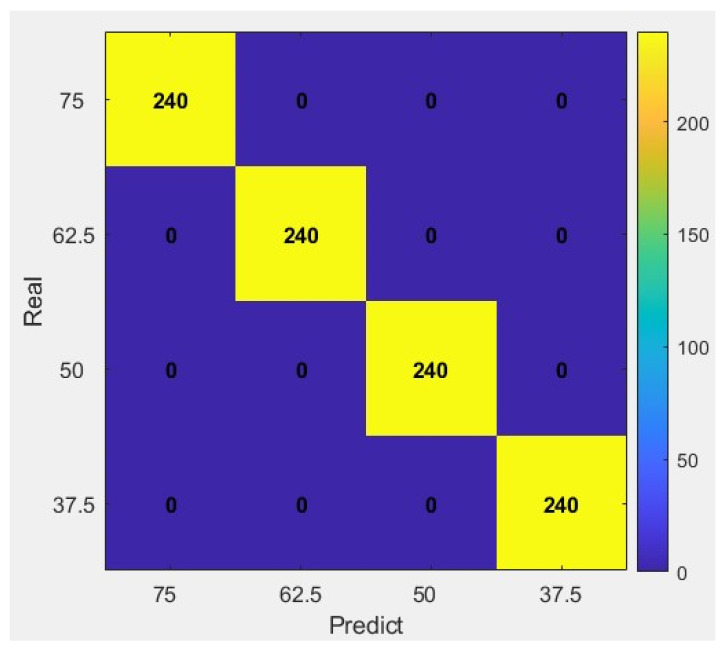
Confusion Matrix of CS-SVM-RFE Detection Results.

To investigate the recognition capability of the model for bolt loosening signals after introducing the cost-sensitive learning mechanism, the vibration signal dataset for each operating condition was expanded to 40 samples through experiments, resulting in a total of 160 samples. The dataset was subsequently divided into training and testing sets at a ratio of 7:3. Using the aforementioned CS-SVM-RFE model, 20 random loosening detection tests were conducted on the pipeline flange bolts. The overall confusion matrix is shown in Figure 16 below.

The model’s detection accuracy for bolt loosening signals under a tightening torque of 37.5 Nm has significantly improved. This result demonstrates that by augmenting the features with Sample Entropy, Coefficient of Variation, and higher-order moment statistics, and introducing a cost-sensitive learning mechanism, the vibration signals under conditions of severe bolt loosening and unstable contact surface pressure distribution can be effectively identified.

**Figure 17:**
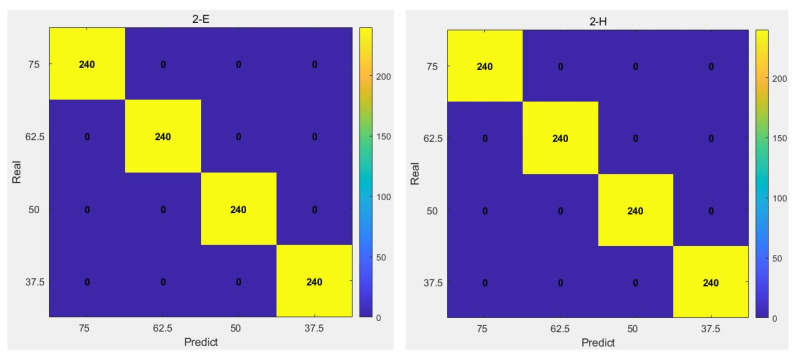
Confusion Matrix for Bolt Loosening Detection at Different Excitation Positions.

To evaluate the bolt loosening detection performance of the system under different excitation positions, excitations were applied at two distinct locations under four loosening conditions of Bolt 2: Point E, which is relatively far from both the sensor and Bolt 2, and Point H, which is relatively close to the sensor. For each loosening condition, 40 data samples were collected. The dataset was divided into training and testing sets with a ratio of 7:3, and the aforementioned model was used for training and testing. The test results are shown in Figure 17.

As indicated by the confusion matrix of the test results, the model maintains good detection performance even when the relative distance between the excitation point and the loose bolt varies.

## 5. Conclusions

This paper addresses the relatively understudied area of bolt loosening detection in gasketed pipeline flanges and adopts a detection method based on the stress wave principle. Systematic experimental investigations and data analyses were conducted on a single test platform consisting of a PN40 DN80 flange, eight M16 bolts, and a spiral wound gasket. The following conclusions are drawn:

(1) Finite element simulations were performed on the normal pressure of the gasket before and after bolt loosening in the flange structure. The analysis reveals that after bolt loosening, the instantaneous elastic properties of the gasket in the loosened region and the opposite region, as well as the overall pressure distribution on the gasket, undergo changes. Moreover, the extent of these changes varies with the degree of loosening. Combined with the principle of wave propagation in the gasket, this provides a theoretical basis for the bolt loosening detection scheme for pipeline flanges.
(2) By comparing the excitation effects of different impact forces and hammer head materials, the optimal detection parameters for this test platform were determined: an impact force of 1000 N and an aluminum alloy hammer head. This parameter configuration excites stress wave signals covering the sensitive frequency band for loosening (0–12 kHz), providing an experimental reference for similar platforms.
(3) Based on a multi-domain feature system, the recognition performance of several models, including SVM, Random Forest, Bagging, and 1D-CNN, was compared. The results demonstrate that SVM-RFE achieves the best overall performance in terms of accuracy (98.44%), stability (standard deviation of ±2.78%), and computational efficiency (0.05 s), validating the effectiveness of combining recursive feature elimination with a support vector machine.
(4) To address the issues of feature dispersion and low recognition accuracy under severe loosening conditions, feature enhancement and a cost-sensitive learning mechanism were introduced. By setting the misclassification cost for the 37.5 Nm category to three times that of the other categories, the recognition accuracy for this condition was effectively improved.
(5) The CS-SVM-RFE model was employed to detect signals under different excitation point locations. The results demonstrate that the model maintains a robust capability for identifying the degree of bolt loosening even when the excitation point is altered.

Nevertheless, this bolt looseness detection method still has certain limitations:

(1) For flange structures with different dimensions, gasket types, bolt quantities, or materials, the dynamic characteristics of the structure and the stiffness distribution of the contact interface will inevitably vary. Consequently, the optimal detection parameters and model performance identified in this study may require recalibration or adjustment.
(2) In practical pipeline flange applications, gasket aging alters the shear modulus and mechanical impedance of the gasket. Additionally, the presence of a medium within the pipeline significantly changes the acoustic boundary conditions of the system. The medium modifies the mechanical impedance at the pipe wall–medium coupling interface, causing stress wave energy to leak from the pipe wall into the medium, thereby accelerating signal attenuation and altering the modal distribution. Under practical conditions, such as gasket aging and medium-filled pipelines, the robustness of this method still needs further verification.
(3) When the location or number of loosened bolts on the flange changes, the propagation paths of stress waves and the number of wave distortions vary, leading to different changes in the characteristic values extracted from the vibration signals acquired by the sensor. These scenarios are not addressed in this study. Future work will introduce transfer learning strategies to rapidly adapt to loosening identification tasks for the remaining bolts based on labeled data from one or a few bolts, thereby maintaining high recognition accuracy while reducing the cost of retraining.

## Figures and Tables

**Table 1:** Contact Settings for Flange Structure.

Contact Body	Target Body	Contact Type	Friction Coefficient
Upper/Lower Flange	Bolt	Frictional Contact	0.2
Gasket Filler Part	Upper/Lower Flange	Frictional Contact	0.12
Lower Flange	Nut	Frictional Contact	0.2
Bolt	Nut	Bonded Contact	None

**Table 2:** Slope of the gasket pressure–strain curve under different preloads.

Bolt Preload	Slope in Loose Bolt Region	Slope in Opposite Tightened Region	Bolt Preload
22,000 N	194.05	194.07	22,000 N
18,333 N	208.21	184.76	18,333 N
14,667 N	255.29	174.33	14,667 N
11,000 N	164.87	165.93	11,000 N

**Table 3:** Feature Analysis Results under Different Loosening Gradients.

Loosening Gradient	75 Nm	62.5 Nm	50 Nm	37.5 Nm
RMS	0.999687	0.999687	0.999687	0.999687
Kurtosis	28.151410	26.872556	32.886893	35.846192
Crest Factor	9.706888	10.575576	10.479362	10.724966
Shape Factor	1.903378	1.746618	1.881413	1.973033
Impulse Factor	18.475878	18.471490	19.716011	21.160709
Spectral Centroid	2934.62 Hz	3095.82 Hz	2717.80 Hz	2639.61 Hz
Spectral Entropy	5.521010	5.406120	5.404457	5.467011
0–750 Hz	0.340960	0.310512	0.321876	0.317694
750–1500 Hz	0.261762	0.204095	0.270129	0.294518
1500–2250 Hz	0.118807	0.095506	0.108825	0.112581
2250–3000 Hz	0.180899	0.288776	0.227051	0.196322
3000–3750 Hz	0.000229	0.000207	0.000204	0.000314
3750–4500 Hz	0.002400	0.002727	0.001571	0.002330
4500–5250 Hz	0.065550	0.061356	0.046452	0.052196
5250–6000 Hz	0.026290	0.033398	0.021727	0.021735
6000–6750 Hz	0.000001	0.000001	0.000001	0.000001
6750–7500 Hz	0.000018	0.000032	0.000021	0.000016
7500–8250 Hz	0.000562	0.000597	0.000321	0.000420
8250–9000 Hz	0.000277	0.000321	0.000278	0.000234
9000–9750 Hz	0.000009	0.000010	0.000007	0.000015
9750–10,500 Hz	0.000297	0.000367	0.000290	0.000272
10,500–11,250 Hz	0.000844	0.000902	0.000567	0.000764
11,250–12,000 Hz	0.001095	0.001194	0.000680	0.000588

**Table 4:** Detection Results for Different Model Combinations.

Detection Method	Mean Accuracy (%)	Standard Deviation (%)	FI	Training Time (s)
SVM-Lasso	97.19%	±3.29	97.5462	0.05
SVM-RFE	98.44%	±2.78	98.5927	0.05
Bag-Lasso	96.56%	±3.67	96.8923	0.50
Bag-RFE	97.19%	±3.29	97.5462	0.54
RF-Lasso	93.44%	±6.54	94.0384	0.15
RF-RFE	96.56%	±3.32	96.9006	0.15
1D-CNN-RFE	95.00%	±3.29	95.3840	0.87
1D-CNN-Lasso	93.75%	±6.87	94.4152	0.82

## Data Availability

The original contributions presented in this study are included in the article. Further inquiries can be directed to the corresponding author.
